# Long-term monitoring and analysis of the longitudinal differential settlement of an expressway bridge–subgrade transition section in a loess area

**DOI:** 10.1038/s41598-022-23829-y

**Published:** 2022-11-11

**Authors:** Yang Du, Xiongjun He, Chao Wu, Weiwei Wu

**Affiliations:** 1 School of Transportation and Logistics Engineering, Wuhan University of Technology, Wuhan, 430063 China; 2 Wuhan Municipal Engineering Design & Research Institute Co., Ltd, Wuhan, 430023 China

**Keywords:** Civil engineering, Hydrogeology

## Abstract

To solve the problem of "bridgehead bumping" in the transition section between the road and bridge of an expressway in a collapsible loess area, a lime–soil compaction pile composite foundation is used for the first time in the transition section between the road and bridge of an expressway in China; the loess subgrade is improved by adding lime, and the subgrade is arranged in a multilayer geogrid for the joint treatment of various engineering measures. At the same time, a new type of precision differential pressure settlement meter is used to monitor the long-term settlement of a bridge–subgrade transition section with a small settlement magnitude after the joint treatment, and the distribution characteristics and variation laws of the settlement along the longitudinal direction of the line are obtained. The results show that the effect is better and the differential settlement is smaller when using a lime–soil compaction pile composite foundation; lime improves the loess subgrade backfill, and the multilayer geogrid addresses the bridge–subgrade transition in the collapsible loess area. The differential settlement and settlement rate of the subgrade and abutment increase with increased monitoring time, and the differential settlement increases gradually, while the growth rate decreases gradually and finally tends to be stable. The differential settlement of the transition section is predicted and analysed by using a hyperbolic curve, exponential curve and their combination in a prediction model, and the prediction analysis shows that the combined prediction model has the best prediction effect. These research results can provide guidance and reference for the design and construction of subgrade structures similar to the wet transition section between roads and bridges.

## Introduction

The bridge–subgrade transition section is the connection area between a bridge and its subgrade. If the treatment process and construction control of the bridge–subgrade transition section are not effective in the project, differential settlement of the bridgehead and the expansion joint (bridgehead approach) can easily occur, which can create steps in the pavement longitudinal slope and cause vehicles to jump when they pass over the joint, a phenomenon known as a "bridgehead bump".

Scholars at home and abroad have carried out extensive research on the differential settlement of bridge-approach transition sections. True^1^ noted that the uneven change of the longitudinal stiffness of the transition section along the line can lead to serious deformation and damage of the transition section, and the stiffness difference and geometric irregularity of the road and bridge can inevitably affect the operational quality of the expressway. Li et al.^2^ studied the factors affecting the deformation and damage of a bridge–subgrade transition section and proposed a quality assessment method for the transition section and treatment measures to reduce the damage. Martin et al.^3^ used software to study the settlement deformation characteristics of a bridge–subgrade transition section under cyclic vehicle loading. Sorasak et al.^4^ found through the long-term monitoring of a bridge transition section near Bangkok that the main reason for the differential settlement of a bridge–subgrade transition section is that the foundation of the bridge and its approach structure are on different soil layers. At the same time, long-term observations have shown that differential settlement can lead to sudden changes in the longitudinal slope and driver discomfort. Scholars such as Jens^5^ have found that long-term cyclic traffic loads can cause plastic cumulative deformation of the track, which leads to irregular track geometry, aggravates track irregularities and affects the normal operation of rail transit. Yang et al.^6^, to address the uneven settlement of a transition section between the road and bridge of a high-speed railway, comprehensively adopted a filling method of mixing graded broken stone with fly ash, cement and roller compacted concrete in the transition section and achieved good results. Liu et al.^7^ found that the accumulative settlement of a bridge–subgrade transition section can be significantly reduced by filling the concrete transition section with rubber. Zhang et al.^8^ found that the uneven settlement of a road–bridge transition section can cause damage to the pavement structure of the transition section, which can seriously affect the operation and maintenance of an expressway. This phenomenon can be alleviated by setting the rigid–flexible transition section. Leng et al.^9^ studied the equivalent stiffness of structures on both sides of a bridge–subgrade transition section and noted that the value of the equivalent stiffness on the side with a smaller stiffness of the transition section is not only related to its own material properties but also related to the material properties on the side with a larger stiffness. Jia et al.^10^ studied the influence of the approach slab, vehicle running speed and subgrade stiffness on the running of a vehicle body through an analysis of the dynamic characteristics of a bridge–subgrade transition section. Hu et al.^11^ proposed two methods to reduce the settlement of bridgehead embankments, namely, mixing loess with sand and reinforcing with a geogrid. Shen et al.^12^ analysed the influence of geogrid reinforcement on the settlement of bridge–subgrade transition sections in loess areas through numerical simulations.

In summarizing the current research results, it can be found that the "bridgehead bump" is caused by the excessive differential settlement of the transition section between the subgrade and the bridge and culvert. The main reasons are as follows: (1) The structural stiffness of the subgrade and the bridge and culvert structure is quite different in the transition section area, which leads to differences in the deformation of the road bridge and the bridge–culvert structure under long-term load, resulting in uneven settlement. (2) After uneven settlement occurs in the transition section, the driving of vehicles produces obvious dynamic action, and the increased dynamic action further increases the uneven settlement. (3) In some special soil areas (such as loess areas), the consolidation settlement of the soil itself is large due to the poor filling of the subgrade and the difficulty of compaction. (4) In some special soil areas (such as soft soil and loess areas), a foundation treatment scheme is not in place, resulting in poor foundation stability and large deformations in later periods.

At present, the main settlement control methods of road–bridge transition sections in collapsible loess areas include composite foundation treatment methods, such as mixing piles, lime–soil compaction piles, and soft foundation replacement. For the settlement control of subgrade filling, high-quality filling (such as gravel, graded sand and stone) or lime and cement can be used to improve poor filling (such as loess, silt, etc.) to control the compactness and stability of subgrade filling. At the same time, polymers, geotextiles and geogrids can be added to the subgrade fill to reduce the differential settlement deformation of the transition section^13–18^. These methods can have a significant effect on the differential settlement control of bridge–subgrade transition sections in collapsible loess areas.

These problems of vehicle bump at bridge head are ultimately the settlement of rock and soil. There are many research methods and many theoretical research and numerical analysis research results on the impact of rock and soil settlement and deformation on the construction of some highway bridge infrastructure. For example, Sun et al.^19,20^ introduced the unified strength theory and used the elastic strain of softening zone and residual zone to analysed the impact of rock and soil displacement on tunnel infrastructure. At the same time, they also studied the failure behavior of the tunnel face caused by the deformation of rock and soil, and studied the influencing factors of the tunnel face stability through parametric research. However, there are also relevant theoretical and numerical simulation analysis and research on the specific study of vehicle bump at bridgehead of expressway, but the research on some relevant engineering examples is not enough, especially the research on the settlement control of vehicle bump at bridgehead in collapsible loess area. There are relatively few relevant studies and data on long-term monitoring of differential settlement of bridge subgrade transition section in loess region, and long-term monitoring of differential settlement of bridge subgrade transition section in loess region treated by engineering measures has not been studied. The lack of monitoring data of long-term engineering cases leads to the lack of long-term experimental data testing of relevant research theories. This hinders the further development of relevant theoretical research and technical application.

Therefore, this paper is the first to use lime–soil compaction pile composite foundations in a road–bridge transition section of the Tianyong Expressway in Gansu Province, China. The subgrade is backfilled with improved loess mixed with lime, and at the same time, multilayer geogrids are arranged for the joint treatment of various engineering measures. This study is the first to monitor the differential settlement of a bridge–subgrade transition section by using a new type of precise differential pressure multipoint profile settlement instrument, and the distribution characteristics and variation laws of the differential settlement along the longitudinal direction of the bridge–subgrade transition section are obtained. The research results provide basic data for follow-up in-depth study of the differential settlement characteristics of a bridge–subgrade transition section. This can also provide guidance and reference for the design and construction of similar bridge–subgrade transition sections.

## Project overview

The monitoring site of the transition section of the Tianyong Expressway is located in Ningxian County, Gansu Province. The construction site is the transition section of a subgrade and bridge. The bridge is the Chengbei River Bridge, located approximately 500 m north of Ningxian County. The total length of the bridge is 920 m, the maximum height of the bridge is approximately 81 m, and the maximum pier height is 73 m. It is a two-way four-lane expressway with a design speed of 80 km/H and a highway level 1 vehicle load. The monitoring work site is the transition section of abutment No. 21, which is a ribbed slab abutment. The height of the abutment is approximately 7 m, and the length of the abutment slab is 8 m. The thickness of the transition section pavement structure is approximately 0.78 m. The subgrade slope is 7 m high, and the slope is set at 1:1.5. The foundation is a self-weight collapsible site, and the collapsibility grade is III–IV (serious ~ very serious).

Considering the lack of high-quality filling materials in collapsible loess areas, to reduce the differential settlement of the transition section, lime–soil compaction piles are used for the first time to treat the composite foundation of the abutment transition section in this test. Lime-improved loess is used to backfill the subgrade soil, and a variety of engineering measures are used to address the challenge by laying a multilayer geogrid to minimize the differential settlement of the transition section.

A lime–soil compaction pile composite foundation is used to treat the foundation of the bridge–subgrade transition section, which is a very practical foundation treatment scheme in collapsible loess areas, and its settlement control effect is also obvious. The lime–soil pile is 8 m long, the pile spacing is 1 m, and the arrangement is square.

The backfill of the transition section subgrade is based on a number of indoor geotechnical tests, data analyses and calculations and finally adopts the scheme of local loess mixed with 5% lime. The soil is taken from the borrowing area of the bridgehead embankment of the Chengbei River Bridge in the TY15 Contract Section of Tianyong Expressway in Gansu Province. According to the field drilling data, the loess in the project section is mainly newly accumulated loess (${\mathrm{Q}}_{4}^{2}$), upper Pleistocene series new loess (${\mathrm{Q}}_{3}^{\mathrm{eol}}$) and upper Pleistocene series eluvial paleosol (${\mathrm{Q}}_{3}^{\mathrm{el}}$). The loess used for subgrade backfilling is the newly accumulated loess (${\mathrm{Q}}_{4}^{2}$), and its physical properties are shown in Table 1. The grain gradation of the loess obtained by the screening test is shown in Table 2. Grade II slaked lime meeting the requirements of the Technical Rules for Construction of Highway Pavement Base (JTG/T F20-2015) is selected.

**Table 1:** Physical properties of loess.

Liquid limit ${\upomega }_{\mathrm{L}}$ (%)	Plastic limit ${\upomega }_{\mathrm{p}}$ (%)	Plasticity index ${\mathrm{I}}_{\mathrm{p}}$	Relative density of soil particles ${\mathrm{G}}_{\mathrm{s}}$	Coefficient of collapsibility	Coefficient of self-weight collapsibility
26.3	16.7	9.6	2.71	0.022	0.01

**Table 2:** Grain size distribution of loess.

Sieve diameter/mm	9.6	4.75	2.36	1.18	0.6	0.3	0.15	0.075
Percent of passing aperture/%	100	94.13	82.39	74.54	62.38	48.64	42.98	28.7

Meanwhile, 3 layers and 4 layers of geogrids are laid on the base and top of the subgrade of the transition section, respectively, and the spacing between the geogrids is 20 cm.

To monitor the differential settlement of the bridge–subgrade transition section, this paper uses a new type of differential pressure high-precision vertical displacement measurement sensor combined with a solar power supply and Internet of Things technology to form a complete set of automatic monitoring systems for subgrade settlement, which realizes the remote real-time online monitoring of the settlement of the transition section of the expressway during operation.

The system consists of a wireless remote subgrade settlement monitoring and safety evaluation system, a JMYC-8110AD differential pressure profile settlement meter (Size: Ø 52 mm × 225 mm; Weight: 0.36 KG Range: 500 mm; sensitivity: 0.01 mm; accuracy: 0.1% FS; working temperature: − 30 °C ~ 85 °C; response frequency: 0.5HZ), PVC protection tube (diameter: Ø75mm), a JMJK-II bus acquisition module V1.0 (accuracy: 0.1% FS; resolution: 0.01% FS) Sampling frequency: 1HZ; Weight: 0.5 KG; Working temperature: − 40 °C ~ 85 °C; Mechanical size: 180 mm × 145 mm × 70 mm), a wireless data transmission module (DTU649: mobile Internet module, GPRS wireless data transmission), a solar panel (power: 100 W; Irradiation intensity: 1000 W/m^2^), a battery (capacity: 36 AH; Rated voltage: 12 V), a remote computer (PC computer).

The differential pressure type profile settlement test system consists of a plurality of differential pressure type profile settlement gauges that are installed at different positions. One of them is used as a reference point, and the vertical displacement change of the measuring point relative to the reference point is obtained by measuring the pressure difference between the measuring point and the reference point. The profile settlement gauge is connected through a liquid pipe, an air pipe, a wire and a pull rope, the air pipe and the liquid pipe are connected to a designated position of a liquid tank, the liquid tank maintains the stability of the liquid level and air pressure, and the wire is connected to an acquisition system for automatic acquisition.

The differential pressure profile settlement gauge is easy to install on site. On the foundation plane to be measured, a continuous installation trench is excavated according to the layout position. A flexible protective pipe with a diameter of approximately 60–70 mm is laid in the trench. A stay wire is reserved in the pipe. Then, the installation trench is filled and compacted to complete the construction of the protective pipe. When the construction conditions meet the test requirements, the assembled multiple differential pressure profile settlement meters in series can be dragged into the protective pipeline by wires. The automatic system is arranged at the end of the protective pipeline, and the settlement meter is connected to the wire to complete the automatic acquisition.

**Figure 1:**
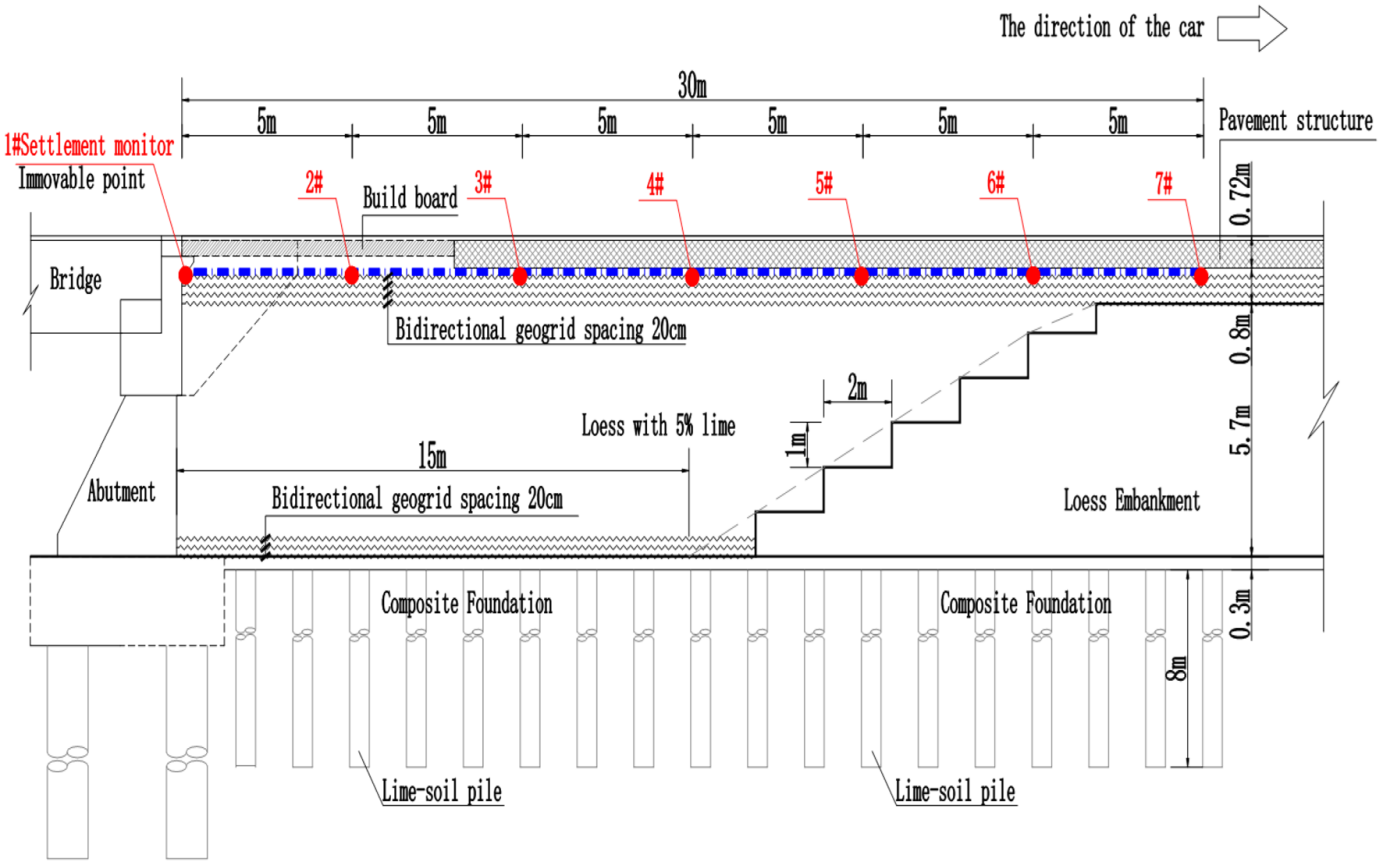
Longitudinal section of the transition section.

**Figure 2:**
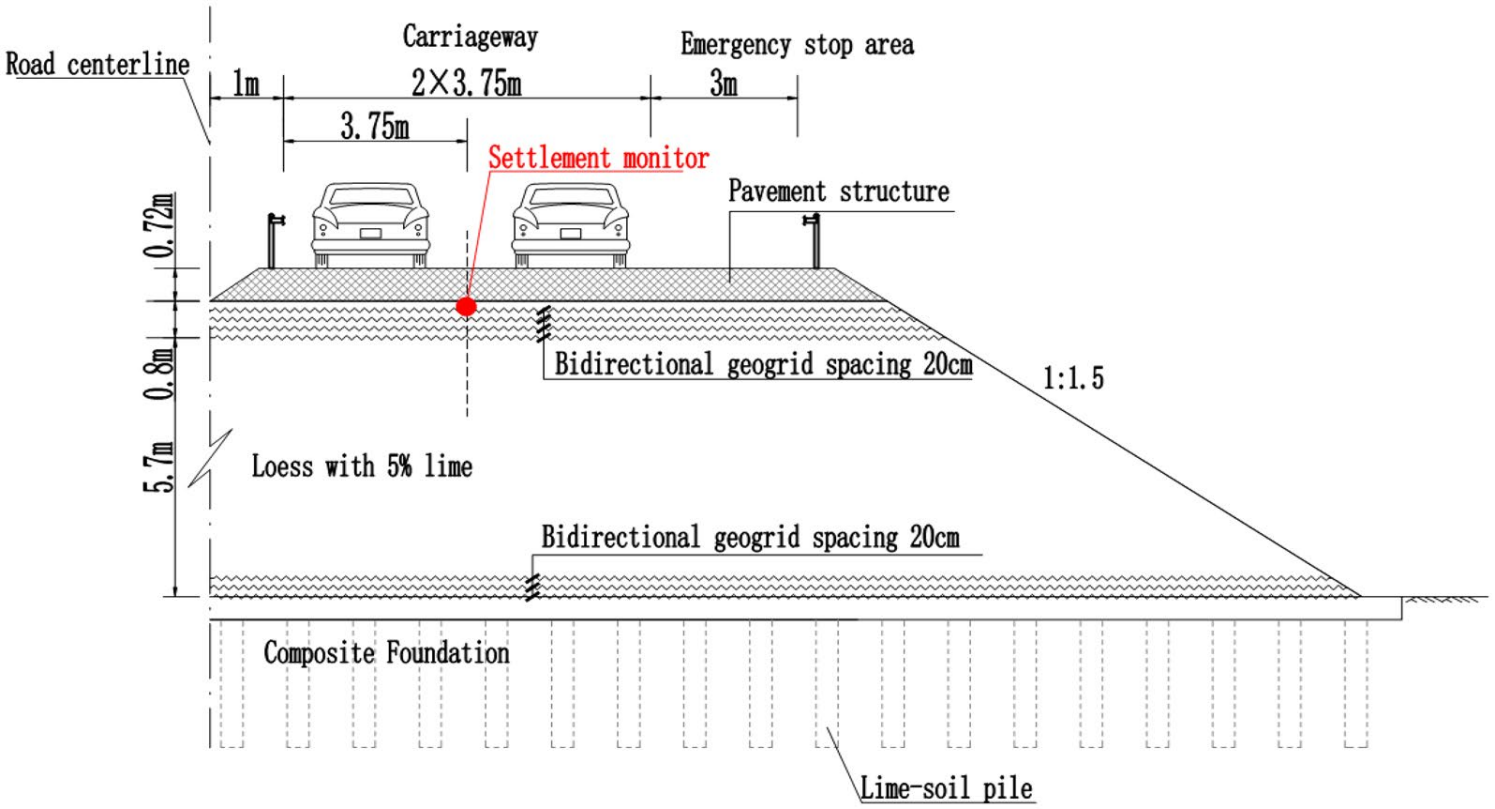
Cross-section of the subgrade.

The treatment method of the road–bridge transition section and the layout of the settlement gauge are shown in Figs. 1 and 2 below.

**Figure 3:**
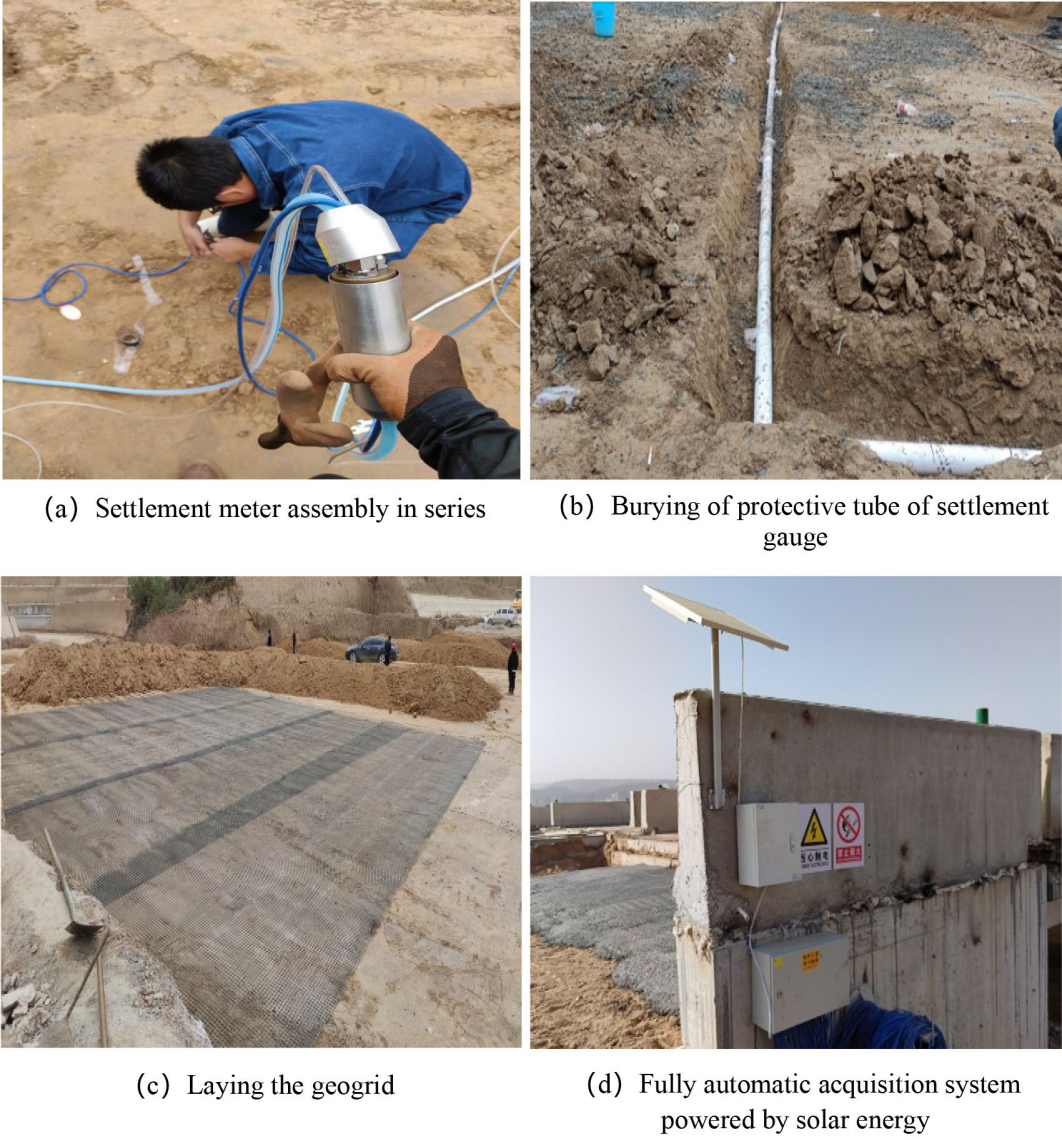
Installation of the profile settlement meter.

In October 2020, the installation of settlement monitoring instruments was carried out on site. During installation, the settlement monitoring fixed point (#1 settlement gauge) was selected under the bracket behind the abutment cap (as shown in Fig. 1 above) and was firmly bound with the abutment through the embedded reinforcement of the abutment structure to ensure that the fixed point (#1 settlement gauge) was fixed. Then, 6 sets of settlement meters were installed at 5 m, 10 m, 15 m, 20 m, 25 m and 30 m away from the back of the abutment. The field installation process is shown in the following Fig. 3.

## Analysis of monitoring data

### Settlement analysis

Since November 2020, the automatic remote monitoring system has been used for real-time monitoring of the bridge–subgrade transition section of the monitoring site for 600 days. At the beginning of monitoring, the sampling frequency was set to 1 time/2 h, and then the data were collected every other day, with a total of 11,130 data points collected. To reduce the vibration impact on the test results caused by many vehicles passing in the daytime, and considering the desolate area of the monitoring site, the wireless transmission data signal is weak from 21:00 to 5:00 at night, and the data cannot be transmitted to the computer remotely, so in order to ensure the integrity of the data, In this study, only the data between 18:00 and 21:00 at night were selected for analysis.

According to the monitoring results, it is very effective to adopt the treatment method of this project: a lime–soil compaction pile composite foundation treatment, combined with a lime loess embankment backfill and a multilayer geogrid to address the abutment transition section in collapsible loess areas. The overall settlement of the abutment transition section is relatively small, and the maximum settlement is less than 5 mm. According to research by relevant scholars on the settlement control index of bridge–subgrade transition sections, considering the vehicle dynamic load coefficient, the maximum settlement of bridge–subgrade transition sections of expressways based on safety should be less than 3 cm^21,22^ through simulation calculations. Therefore, the settlement control effect of the treatment scheme in this project can meet the expectation.

**Figure 4:**
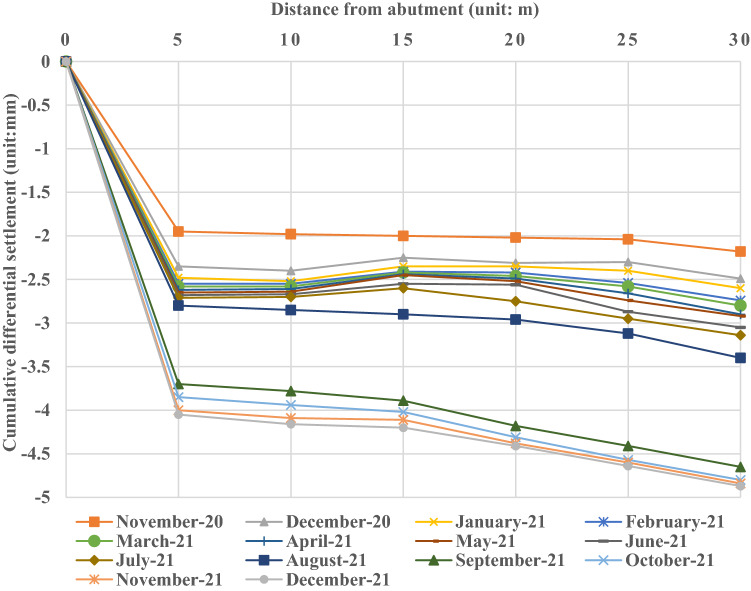
Cumulative differential settlement of monitoring points.

According to the statistical data, the distribution curve of differential settlement for the transition section along the longitudinal direction of the line is obtained, as shown in Fig. 4.

Figure 4 shows that the differential settlement of the bridge transition section gradually increases with increasing distance from the abutment and continuously increases with increasing time. During the monitoring period, the maximum settlement of the transition section is approximately 4.8 mm, and the minimum settlement is 1.9 mm. The change trend of differential settlement at each measuring point along the longitudinal direction of the line in the transition section is relatively consistent; the area with the most obvious change is mainly concentrated 15 m ~ 20 m away from the abutment, and there is a settlement difference of nearly 0.5 mm between the two corresponding measuring points. It can be preliminarily determined that the subgrade structure within 15 m ~ 20 m from the abutment is the area with the most significant settlement in the transition section.

**Figure 5:**
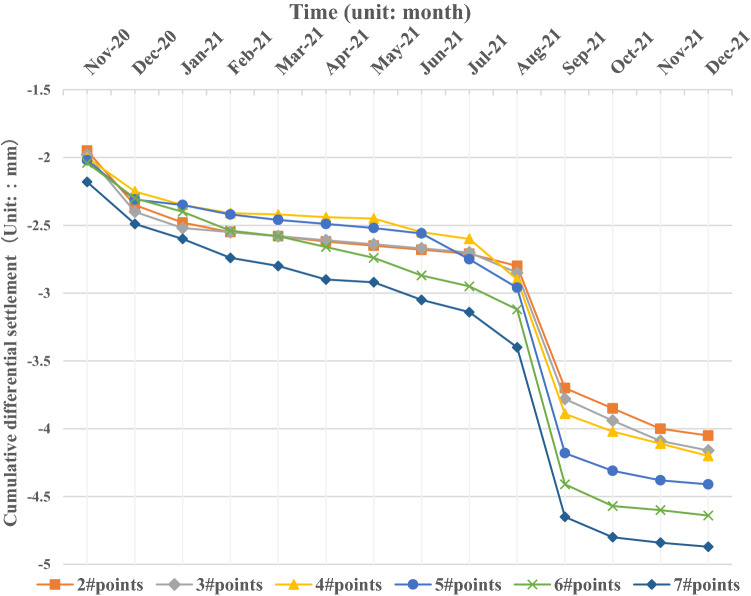
Cumulative differential settlement of monitoring points.

It can also be seen in Fig. 5 that the differential settlement of each monitoring point relative to the fixed point of the abutment continues to increase in the range of 2.0 mm ~ 4.8 mm, and the farther away from the abutment, the greater the differential settlement and its change amplitude. At the same time, in the initial stage of monitoring, the bridge transition section has not yet been opened to traffic; only one month later, there is a 2.0 mm ~ 2.2 mm differential settlement between each monitoring point and the fixed point of the abutment, and it is a differential settlement between the whole transition section and the fixed point, indicating that the transition section has an instantaneous settlement at the beginning. In August 2021, after the whole expressway was officially opened to traffic, the settlement of each monitoring point increased significantly in a month, and the overall settlement increased by almost 1.0 mm ~ 1.5 mm. Under the influence of vehicle loads, the main consolidation settlement occurred in the transition section. After that, the differential settlement of the transition section increases gradually, and the growth rate is larger at the beginning of operation or in the early stage and then decreases significantly in the later stage, which indicates that under the long-term cyclic action of vehicle loads, the subgrade of the transition section has a certain cumulative deformation, and the settlement trend becomes slower.

### Settlement rate analysis

**Figure 6:**
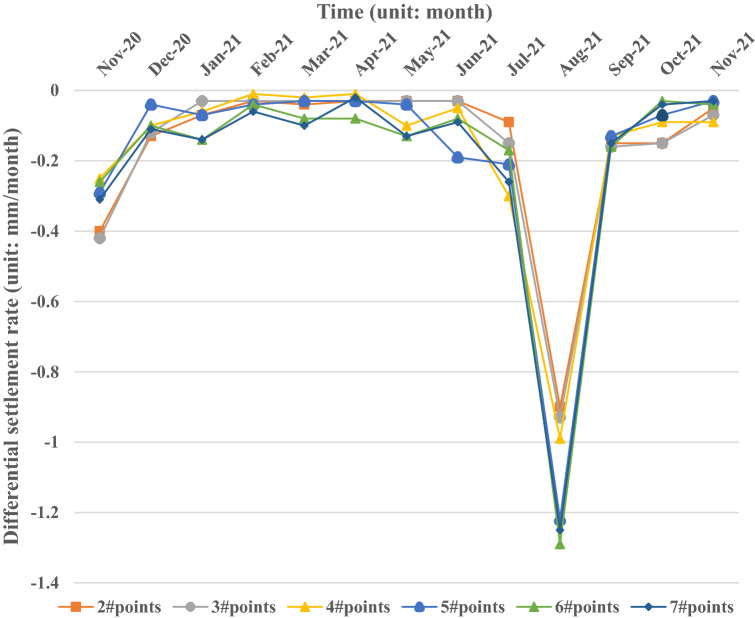
Cumulative differential settlement and settlement rate change diagram of monitoring points.

To study the variation law of differential settlement growth with time, the statistical data of monthly differential settlement are compared from the beginning to the end, and the settlement of the transition section structure is reflected by the amount of settlement in a month. The settlement rate of each monitoring point in the 13 months of the test is shown in Fig. 6 below.

Figure 6 shows that the settlement rate varies greatly with the distance from the abutment, which is similar to the longitudinal distribution of settlement along the line. At the same time, after August 2020, with the increase in vehicle running load on the expressway, the settlement rate has a rapid increase, then gradually decreases, and finally tends to be stable. In November 2021, when the vehicles have been running for 4 months, the differential settlement rate enters a stable state. With the continuous increase in cumulative load, the settlement rate of each measuring point finally stabilized in the range of 0.06 mm/month.

## Settlement prediction analysis

For a loess subgrade, the deformation process is complex, and it is difficult to accurately calculate the development of this kind of settlement using analytical methods. It is faster and more reliable to calculate the development law of settlement with time by fitting the measured data of field settlement. Domestic and foreign scholars have carried out considerable research on fitting prediction models for soil settlement, and their fitting prediction methods mainly include the exponential curve method, hyperbolic method, Asaoka method and Poisson curve method. Among them, the exponential curve method and hyperbolic method are simpler than other prediction methods; because they are convenient for engineers to apply and can adapt to the fitting of most subgrade settlement curves, they are widely used in the settlement prediction of highway subgrades, and the methods are relatively mature and reliable^23,24^. In recent years, the creep settlement prediction method has become a predominant prediction method because it has good applicability for newly filled soil. The GM method and BP neural network prediction method based on neural networks^25,26^ are self-adaptive and suitable for situations with less measured settlement data.

At present, the settlement prediction method of the related research is generally only suitable when the overall settlement magnitude of ordinary soil, new filling soil, new subgrade, or soft soil subgrade is relatively large. However, for a collapsible loess area, especially a roadbed–bridge transition section after better subgrade treatment filled with lime-improved loess and a layered geogrid to reduce the settlement deformation, the magnitude of the later settlement change is small, which is different from the settlement law of the subgrade in general, so research on an applicable prediction model in this case is limited.

Therefore, considering the settlement mechanism of the subgrade structure of Tianyong Expressway and the applicable conditions of various prediction models, based on the settlement fitting methods of exponential curves and hyperbolas^21–27^, which are widely and maturely used in the industry at present, and according to the requirements of the optimal target fitting of settlement fitting, this paper proposes a new combined prediction model to fit the settlement of the transition section of Tianyong Expressway.

The expression for the hyperbolic model is:

1 $${y}_{1}\text{=}{a}_{1}+\frac{t-{b}_{1}}{{c}_{1}-{d}_{1}\times t}$$

The expression for the exponential model is:

2 $${y}_{2}={a}_{2}-{b}_{2}\times {e}^{\left(-{c}_{2}\times t\right)}$$

The expression of the new combination model formed after combination is:

3 $${y}_{3}={y}_{1}^{{n}_{1}}+{y}_{2}^{{n}_{2}}$$

where ${a}_{1}$, ${a}_{2}$, ${b}_{1}$, ${b}_{2}$, ${c}_{1}$, ${c}_{2}$, ${d}_{1}$ and ${n}_{1}$, ${n}_{2}$ are the corresponding fitting parameters, and $t$ is the number of monitoring days.

To test the accuracy of the prediction model, the common accuracy and reliability indices in the engineering field can be adopted: goodness of fit (${R}^{2}$), sum of absolute error squares (SSE) and mean absolute percentage error (MAPE). The specific formula is as follows:

1. Goodness of fit (${R}^{2}$)
  When the data is counted, The sum of squares of residuals is: $ESS=\sum {\left({y}_{t}-\widehat{{y}_{t}}\right)}^{2}$, Regression sum of squares $RSS=\sum {\left({y}_{t}-\overline{{y }_{t}}\right)}^{2}$, $TSS=RSS+ESS$, Fit the correlation coefficient ${R}^{2}=\frac{RSS}{TSS}$, The correlation coefficient ${R}^{2}$ is between 0 and 1.
2. Sum of squared absolute error (SSE):
  4 $$SSE = \sum\limits_{i = 1}^{n} {\left( {S_{p} - S_{m} } \right)^{2} }$$
3. Mean absolute percent error (MAPE):
  5 $$MAPE = \frac{1}{n}\sum\limits_{i = 1}^{n} {\left( {\frac{{\left| {S_{p} - S_{m} } \right|}}{{S_{p} }}} \right)} \times 100$$
  where ${S}_{p}$ is the predicted value and ${S}_{m}$ is the monitored value. The smaller the MPAE value is, the better, generally less than 10%, which reflects that the prediction accuracy of the model is higher.

After the expressway was opened to traffic in August 2021, according to the settlement monitoring data, it was found that there was a significant increase in settlement within a month, and then it tended to be stable. Therefore, the model fitting data take the daily monitoring data of 200 days from September 1, 2021, to March 20, 2022. Through the curve fitting of the monitoring data, a prediction model suitable for the differential settlement of the bridge–subgrade transition section of an expressway in similar loess areas is selected through analysis and research.

Tables 3, 4, 5, 6, 7 and 8 show that, from the inspection indicators, the three curve models can meet the requirements for fitting the relationship between the settlement of the bridge–subgrade transition section of the project and the vehicle running load. According to the accuracy index (R^2^) and the stable reliability index (SEE and MPAE) of the evaluation prediction model, the fitting effect of the combination prediction model is the best, the exponential model is the second best, and the hyperbolic model is slightly worse. The shows that the combined forecasting model is better for the similar situation of this project.

**Table 3:** Cumulative differential settlement prediction model of monitoring point 2.

Prediction model	Parameters of the equation	R^2^	SSE	MPAE (%)
Hyperbolic model	${a}_{1}$ = 5.57, ${b}_{1}$ = 1.45, ${c}_{1}$ = 61.24, ${d}_{1}$ = 0.21	0.905	0.011	9.3
Exponential curve model	${a}_{2}$ = 6.14, ${b}_{2}$ = 2.04, ${c}_{2}$ = 0.00195	0.915	0.008	7.3
Combined forecasting model	${n}_{1}$ = 0.79, ${n}_{2}$ = 0.26	0.945	0.007	4.3

**Table 4:** Cumulative differential settlement prediction model of monitoring point 3.

Prediction model	Parameters of the equation	R^2^	SSE	MPAE (%)
Hyperbolic model	${a}_{1}$ = 3.57, ${b}_{1}$ = 2.25, ${c}_{1}$ = 21.51, ${d}_{1}$ = 0.32	0.902	0.012	10.3
Exponential curve model	${a}_{2}$ = 3.14, ${b}_{2}$ = 0.04, ${c}_{2}$ = 0.085	0.938	0.006	5.7
Combined forecasting model	${n}_{1}$ = 0.52, ${n}_{2}$ = 0.75	0.958	0.006	3.5

**Table 5:** Cumulative differential settlement prediction model of monitoring point 4.

Prediction model	Parameters of the equation	R^2^	SSE	MPAE (%)
Hyperbolic model	${a}_{1}$ = 4.95, ${b}_{1}$ = 0.85, ${c}_{1}$ = 101.51, ${d}_{1}$ = 0.73	0.914	0.009	9.1
Exponential curve model	${a}_{2}$ = 5.14, ${b}_{2}$ = 3.07, ${c}_{2}$ = 0.025	0.941	0.007	3.7
Combined forecasting model	${n}_{1}$ = 0.83, ${n}_{2}$ = 0.13	0.952	0.005	3.7

**Table 6:** Cumulative differential settlement prediction model of monitoring point 5.

Prediction model	Parameters of the equation	R^2^	SSE	MPAE (%)
Hyperbolic model	${a}_{1}$ = 6.12, ${b}_{1}$ = 85.12, ${c}_{1}$ = 11.25, ${d}_{1}$ = 0.86	0.944	0.009	7.1
Exponential curve model	${a}_{2}$ = 5.34, ${b}_{2}$ = 2.37, ${c}_{2}$ = 0.037	0.944	0.006	6.7
Combined forecasting model	${n}_{1}$ = 0.59, ${n}_{2}$ = 0.48	0.967	0.005	2.2

**Table 7:** Cumulative differential settlement prediction model of monitoring point 6.

Prediction model	Parameters of the equation	R^2^	SSE	MPAE (%)
Hyperbolic model	${a}_{1}$ = 1.12, ${b}_{1}$ = 20.31, ${c}_{1}$ = 6.65, ${d}_{1}$ = 0.136	0.901	0.013	11.2
Exponential curve model	${a}_{2}$ = 7.51, ${b}_{2}$ = 15.37, ${c}_{2}$ = 0.008	0.922	0.009	6.9
Combined forecasting model	${n}_{1}$ = 0.73, ${n}_{2}$ = 0.34	0.953	0.007	4.1

**Table 8:** Cumulative differential settlement prediction model of monitoring point 7.

Prediction model	Parameters of the equation	R^2^	SSE	MPAE (%)
Hyperbolic model	${a}_{1}$ = 0.82, ${b}_{1}$ = 43.75, ${c}_{1}$ = 8.14, ${d}_{1}$ = 0.11	0.951	0.005	2.8
Exponential curve model	${a}_{2}$ = 5.15, ${b}_{2}$ = 102.36, ${c}_{2}$ = 0.027	0.954	0.004	2.3
Combined forecasting model	${n}_{1}$ = 0.37, ${n}_{2}$ = 0.82	0.964	0.004	2.2

To verify the accuracy of the prediction model, the above three prediction models are used to predict the settlement of the bridge–subgrade transition section on June 20, 2022 (the 300th day), when the expressway has been open to traffic for 10 months. The comparison between the model prediction results and the actual field monitoring values is shown in Table 9.

**Table 9:** Analysis of cumulative differential settlement prediction model of monitoring points.

Monitoring point	Measured value (mm)	Hyperbolic model		Exponential curve model		Combined forecasting model	
		Predicted value (mm)	Error (%)	Predicted value (mm)	Error (%)	Predicted value (mm)	Error (%)
#2	− 4.35	− 4.58	− 5.29	− 4.15	4.60	− 4.13	5.06
#3	− 4.16	− 4.63	− 11.30	− 4.31	− 3.61	− 4.33	− 4.09
#4	− 4.1	− 4.47	− 9.02	− 4.18	− 1.95	− 4.22	− 2.93
#5	− 4.41	− 4.71	− 6.80	− 4.39	0.45	− 4.47	− 1.36
#6	− 4.64	− 5.01	− 7.97	− 4.27	7.97	− 4.79	− 3.23
#7	− 4.87	− 5.14	− 5.54	− 4.36	10.47	− 4.78	1.85

It can be seen in Table 9 above that the predicted value of the hyperbolic model is larger than the measured value, and the relative error range between them is − 5.54% to 11.30%. The predicted values of the exponential curve model were smaller than the measured values, and their relative errors ranged from 10.47 to − 3.61%. The predicted values of the combination forecasting model are close to the measured values, and their relative error ranges from − 4.09 to 5.06%. The overall error of the combined forecasting model is the smallest, and the degree of coincidence with the measured value is the highest.

In this project, the subgrade of a bridge–subgrade transition section is treated by a lime–soil compaction pile composite foundation combined with lime-improved loess and a multilayer geogrid; the differential settlement is relatively small, the prediction effect of the combination prediction model is the best, and the prediction error of the exponential curve model and hyperbolic model is relatively large.

## Conclusion

To solve the problem of differential settlement in bridge–subgrade transition sections of expressways in collapsible loess areas, lime–soil compaction pile composite foundations are used for the first time in this paper. Combining the improvement of loess by adding lime with the arrangement of multilayer geogrids, a variety of engineering measures are used to minimize the occurrence of differential settlement. In this paper, the differential settlement of a bridge–subgrade transition section is monitored for a long time by using a new type of differential pressure profile settlement instrument, and the monitoring results of settlement, settlement rate and their distribution characteristics and variation along the longitudinal direction of the line are analysed. Three prediction models are used to predict the differential settlement of the bridge–subgrade transition section, and the following conclusions are drawn:

1. According to the monitoring results, the effect of using a lime–soil compaction pile composite foundation combined with a loess embankment backfill mixed with lime and adding a multilayer geogrid to deal with the transitional section of an abutment in a collapsible loess area is very obvious; the overall settlement and differential settlement of the transitional section of the abutment are relatively small, and the effect of controlling the settlement of the treatment scheme meets expectations. The relevant treatment methods can be applied in similar projects.
2. At the initial stage of construction completion, a relatively obvious instantaneous settlement occurs in the transition section of the road and bridge, and the settlement is approximately 2 mm. At the initial stage after expressway operation, a relatively obvious primary consolidation settlement occurs under the action of vehicle loads, and the settlement is approximately 1.5 mm.
3. During the monitoring period, the maximum settlement of the transition section at the bridge end and the subgrade structure is 4. 87 mm, and the minimum settlement is 1.9 mm. The subgrade structure within 15–20 m from the abutment is the most significant area of the transition section settlement.
4. The differential settlement of the transition section on both sides of the bridge increases gradually with increasing distance from the abutment and with increasing operation time (cumulative vehicle load) of the expressway.
5. During the monitoring period, the settlement rate of each measuring point in the transition section varies within a range of 0.06 mm/month, and the farther away from the abutment, the greater the settlement rate and its variation range. With the increase in the cumulative vehicle load, the settlement rate gradually decreases and finally tends to stabilize.
6. For the settlement of an expressway bridgehead subgrade structure with relatively small settlement, the combined prediction model can better predict its long-term settlement deformation, and the prediction errors of the exponential curve model and hyperbolic model are relatively large.

In addition, due to the complexity of the actual site conditions of the project, there are many factors affecting the differential settlement of the transition section, such as temperature, climate, traffic load level, and subgrade height. This project is limited by the technical level of the monitoring instruments, project site conditions, and economic and other reasons, so it is impossible to study and consider more influencing factors. Further study in this area should be strengthened in the future.

## Data Availability

Some or all data that support the findings of this study are available from the corresponding author upon reasonable request.

## References

[1] Trueh Z (1999). Vehicle-track dynamics on a ramp and on the bridge: Simulation and measurements. Vehicle Syst. Dyn..

[2] Davis D (2020). Transition of railroad bridge approaches. J. Geo Tech. Geo Environ. Eng..

[3] Maringr, Y. Bridge Abutments modelling for seismic response analysis. In *Proceedings of the 4th Cal trans Seismic Research Workshop*. (California Department of Transportation, 1996).

[4] Seawsirikul S, Chantawarangul K (2015). Evaluation of differential settlement along bridge approach structure on Soft Bangkok clay. Geotech. Saf. Risk..

[5] Nielsen JCO, Li X (2018). Railway track geometry degradation due to differential settlement of ballast/subgrade e numerical prediction by an iterative procedure. J. Sound Vib..

[6] Yang C, Tong X (2018). A new design of bridge-subgrade transition sections applied in Beijing–Shanghai high-speed railway. Complexity.

[7] Liu X, Liu P (2016). Feasibility analysis on application of modified concrete contains rubber powder of straddle type monorail train waste tire. Procedia Environ. Sci..

[8] Zhang Y, Li R (2022). The cooperative control of subgrade stiffness on symmetrical bridge–subgrade transition section. Symmetry.

[9] Leng W, Zhou S (2021). Monitoring analysis and prediction of longitudinal differential settlement of track structure in bridge–subgrade transition section of existing heavy haul railway. J. China Rail..

[10] Jia L, Liang R (2017). Experimental study on post-construction settlement monitoring of roadbed-subgrade transition section. J. Rail. Eng..

[11] Hu Y, Shen J, Zhao J (2013). Experimental study on engineering properties of sand loess reinforced by geogrid. Rock Soil Mech..

[12] Shen J, Zhang J, Zhao J (2014). Stress analysis and calculation of adjacent embankment at bridgehead reinforced by geogrid. J. Zhengzhou Univ..

[13] Jiang Y, Li Q, Yi Y, Yuan K, Deng C, Tian T (2020). Cement-modified loess base for intercity railways: Mechanical strength and influencing factors based on the vertical vibration compaction method. Materials..

[14] Gu K, Chen B (2020). Loess stabilization using cement, waste phosphogypsum, fly ash and quicklime for self-compacting rammed earth construction. Constr. Build. Mater..

[15] Yang B, Weng X, Liu J, Kou Y, Jiang L, Li H, Yan X (2017). Strength characteristics of modified polypropylene fiber and cement-reinforced loess. Cent. South Univ..

[16] Zhang J, Li J, Wang J, Xu S (2021). Characteristics of the interface between bamboo grids and reinforced soil of high-filled embankments in loess areas. Adv. Civil Eng..

[17] Luo L, Wang X (2022). Laboratory experiments and numerical simulation study of composite-material-modified loess improving high-speed railway subgrade. Polymers.

[18] Song J, Ma J, Li F (2021). Study on fractal characteristics of mineral particles in undisturbed loess and lime-treated loess. Materials.

[19] Sun Z, Zhang D (2022). Analytical solutions for deep tunnels in strain-softening rocks modeled by different elastic strain definitions with the unified strength theory. Sci. China Technol. Sci..

[20] Sun Z, Zhang D (2022). Model test and numerical analysis for the face failure mechanism of large cross-section tunnels under different ground conditions. Tunn. Undergr. Space Technol..

[21] Chen J, Feng Z (2013). Control standard for differential settlement of bridge–subgrade transition section based on safety. Highw. Transp. Sci. Technol..

[22] Chen X, Zhi X (2006). Research on controlling criterion for differential settlement of bridge approach. Highw. Transp. Sci. Technol..

[23] Zhang X, Li H, Feng X, Chen Z (2012). Research on the prediction of high embankment settlement based on the real-time monitoring on-site. Appl. Mech. Mater..

[24] Xie L (2012). Comparative study on settlement prediction of railway subgrade on collapsible loess area. Appl. Mech. Mater..

[25] Luo JH, Wu C, Liu XL (2018). Prediction of soft soil foundation settlement in Guangxi granite area based on fuzzy neural network model. IOP Conf. Ser. Earth Environ. Sci..

[26] Su XX, Cao YQ, Wen YP (2020). Long-term settlement prediction analysis of overhead transmission tower on recent fill. Ind. Constr..

[27] Feng QZ, Huang T (2020). Application of gray BP neural network combination model in dam settlement monitoring. J. Gansu Sci..

